# TNF-alpha: Roles in pathogenesis and therapeutics in cancer

**DOI:** 10.1016/j.jbc.2026.111251

**Published:** 2026-02-04

**Authors:** Miriam Valenzuela Cardenas, Theodore Scott Nowicki

**Affiliations:** 1 Division of Pediatric Hematology-Oncology, Department of Pediatrics, University of California Los Angeles, Los Angeles, California, USA; 2 Department of Microbiology, Immunology, and Molecular Genetics, University of California Los Angeles, Los Angeles, California, USA; 3 Jonsson Comprehensive Cancer Center, University of California Los Angeles, Los Angeles, California, USA; 4 Eli and Edythe Broad Center for Regenerative Medicine and Stem Cell Research, University of California Los Angeles, Los Angeles, California, USA; 5 Molecular Biology Institute, University of California Los Angeles, Los Angeles, California, USA

**Keywords:** TNF-alpha, T-cells, cancer, inflammation, signaling

## Abstract

Tumor necrosis factor alpha (TNF-α) is a pleiotropic cytokine that can both facilitate tumor progression and directly mediate tumor cell killing. This dual role creates a conundrum in which TNF can be either beneficial or detrimental for a tumor, depending on the context. Herein, we describe the history of the cytokine, the cases in which TNF-α has been considered as a cancer immunotherapy, and the toxicities that can manifest from its use. We also add context to its activity, particularly in T cells (*via* the engagement of TNF receptors) as well as the epigenetic and immunoregulatory pathways that are elicited. Furthermore, we highlight the fundamental differences in the transcriptional and translational regulation of this cytokine, which plays a significant role in the context of malignancy and potential success of immunotherapies. This review aims to provide insight and background on molecular switches, cellular context, and TNF receptor dynamics that determine the role of TNF-α as both tumor suppressor and promoter in different models, which is essential for deriving maximal benefit from TNF therapies.

## History of tumor necrosis factor-alpha and establishment of tumor necrosis factor superfamily

Inflammation has been considered not only a hallmark of cancer, characterized by the release of proinflammatory cytokines from the tumor and its microenvironment, but has also been implicated in the progression of cancer itself (1). One proinflammatory cytokine implicated in this phenomenon is tumor necrosis factor alpha (TNF-α), a multifaceted cytokine originally termed as an endotoxin upon its discovery in the 1960s. Formerly known as “Coley’s mixed toxins,” TNF-α was hypothesized to be a bacterial byproduct that had the ability to regress tumors. Later, it was demonstrated that the tumor regression was independent of bacterial influence and was actually a result of a signaling molecule produced by immune cells as a response to the bacterial insult that led to tumor necrosis (2, 3, 4, 5). Finding the cellular source of TNF-α was of importance, as this could provide insight into its regulation and how it could be harnessed as a therapeutic. Groups eventually determined that one cellular source of TNF-α was macrophages, which was consistent within the context of previous research showing that bacterial infection triggered TNF-α production (3). However, it was subsequently shown that TNF could also be produced by B cells. Although the two factors originating from different cell sources were considered to be effectively synonymous, subsequent studies demonstrated only 30% amino acid homology between the two molecules from the macrophages and B cells (6, 7). The existence of a common surface receptor led to their renaming as TNF-α and TNF-β, respectively. These are distinguished by their cellular source (TNF-α commonly being produced by macrophages and monocytes, whereas TNF-β is commonly produced by activated T and B lymphocytes and NK cells) as well as their overall purpose and regulation (8, 9). Previous research determined that malignant cell lines had the ability to produce both cytokines. However, cell lines that produced TNF-β mRNA were able to secrete the cytokine. Conversely, cell lines producing TNF-α mRNA did not necessarily release it, suggesting that TNF-α gene expression may be controlled predominantly at the post-transcriptional level (9, 10, 11). Subsequent work has gone into understanding how both cytokines differ, and how they both play a role in cancer progression and regression considering their cytotoxic abilities. TNF-α plays a dual role in cancer progression and regression, whereas the activity of TNF-β in cancer has mostly been associated with influencing growth, such as promoting proliferation and mediating antiproliferative effects in different tumor cell lines (9). While both cytokines play an important role in the context of cancer, the scope of this review will focus on TNF-α activity in particular. Research on these cytokines revolutionized the field of cancer immunology research and provided hope for the use of this cytokine as a therapeutic. However, the decades since its discovery were spent unraveling the multiple ways in which TNF-α plays a role in cellular processes, and the intricacies in which TNF-α can act as both a protumor and antitumor agent, with multifaceted roles in mediating apoptosis, proliferation, survival, hematopoiesis, and cell differentiation (12).

TNF-α was subsequently shown to be a multifunctional cytokine normally expressed as a type II transmembrane protein, proteolytically cleaved into its soluble form by TNF-α converting enzyme with the ability to signal through two different receptors, which are differentially expressed on many cell types. Tumor necrosis factor receptor 1 (TNFR1) is expressed on virtually all nucleated cells, whereas TNFR2 is present on specific subsets of cell types, including immune cell subsets, such as regulatory T cells (Tregs), CD8+ T cells, myeloid-derived suppressor cells (MDSCs), as well as endothelial cells and fibroblasts (13). TNFR1 contains a prodeath signaling domain, whereas TNFR2 is mostly associated with preventing apoptosis and a survival gene/protein expression profile (14, 15, 16). Depending on the form that TNF-α is in, it can trigger different cellular processes. For example, while both receptors have the ability to be activated by both soluble and membrane-bound TNF-α, TNFR1 demonstrates a preference for the soluble form of TNF-α, whereas TNFR2 is only fully activated by the membrane-found form of the cytokine (17, 18). One cellular process that is exclusive to TNF-α is its ability to be directly cytolytic (19, 20, 21). Cellular apoptosis can be triggered by a variety of pathways, including cellular damage, the subsequent activation of procaspases and caspases that lead to collapse of the cytoskeleton, disassembly of the nuclear envelope, and fragmentation of DNA (22).

TNF-α can trigger apoptosis by binding to receptors belonging to the TNF superfamily, most notably TNFR1, DR3, FAS-R (CD95), TNF-related apoptosis–inducing ligand receptor 1 (TRAIL-R1, also known as DR4), and TRAIL-R2 (also known as DR5) (23). These receptors can bind TNF-α directly (*e.g*., TNFR1) or can bind another member of the TNF superfamily, such as Fas ligand or TRAIL. Fas ligand is predominantly found on activated T cells, B cells, and NK cells, and to a much lesser extent on dendritic cells and tumor cells. Its expression is tightly regulated and transcriptionally controlled by factors, such as NF-κB and AP-1, as well as post-transcriptional regulation by microRNAs (24). TRAIL is commonly found on various immune cells, such as NK cells, macrophages, dendritic cells, and T cells, mostly in the context of activation and significantly enhanced after exposure to interferon-gamma (25). Apoptosis, in the context of these receptors, is triggered by the presence of death domains. TNF-associated death domain, along with Fas-associated death domain and receptor-interacting protein (RIP), are intracellular, cytoplasmic components that form a complex and trigger caspase activation (26). Caspase activation is a tightly regulated process that requires the building of a death signaling complex and requires multiple forms of activation; otherwise, the process is aborted through the induction of cellular inhibitor of apoptosis proteins (cIAPs) (27, 28). In addition to caspase activation, signaling through any of these proapoptotic receptors leads to transcription factor activation. The cellular response to TNF-α binding to TNFR1 (or any of the death receptors) is to modulate cellular activity by activating canonical NF-κB pathways. However, unsuccessful NF-κB transcription subsequently leads to the development of the death signaling complex, composed of TNF receptor–associated factor (TRAF2), TNF-associated death domain, and RIP proteins, followed by cytoplasmic apoptotic complex II leading to caspase activation (29). In other contexts, when the steps leading to apoptotic cell death are interrupted or not successful, the cell can undergo cellular necrosis (termed necroptosis) following TNF-α exposure. In this case, necroptosis is commonly associated with the rapid lysis of cells and the release of danger-associated molecular patterns, causing an augmentation of the inflammatory response taking place. However, in the context of TNF-α exposure (which already implies a highly inflammatory setting), necroptosis acts as a fast-acting way to get rid of these highly transcriptionally regulated inflammatory cells and therefore, diminish the robust inflammatory reaction (30). TNF-α can also lead to necrosis directly, which is a more unregulated process by virtue of the loss of cell membrane integrity. In these cases, TNF-α can lead to necrosis through the induction of NOX1 (a nonmitochondrial NADPH oxidase), which leads to the production of reactive oxygen species that can cause necrotic cell death (31).

TNFR2 signaling is most associated with protective and anti-inflammatory functionalities. However, its activity is more complex. For example, TNFR2 has been suggested to activate TNFR1 pathways by sequestering TRAF2–cIAP1/2 complexes that induce canonical NF-κB activation, therefore antagonizing TNFR1 activation in the process and leading to cell death (32, 33). Furthermore, TNFR2 can trigger pathways involved in survival and cell migration directly through its activation of noncanonical NF-κB signaling. In addition, TNFR2 has been suggested to play a role in CD8+ T-cell activation, influencing costimulation, cell viability, and enhancing T-cell receptor (TCR)–induced apoptosis (34).

## TNF-α as an immunotherapy and toxicities

Research heavily focused on the mechanistic intricacies of TNF-α signaling and its therapeutic potential, particularly after the observed necrosis it caused in tumors. *In vitro* studies demonstrated its ability to inhibit growth and induce cytotoxicity in multiple malignant cell lines. In addition, *in vivo* studies showed that delivery of this cytokine decreased tumor progression and promoted tumor clearance (35, 36, 37, 38). This suggested that TNF-α could be a promising therapeutic candidate alone or in combination with other agents to improve clinical outcomes. Indeed, preclinical studies showed that systemic TNF-α was able to improve certain conventional chemotherapeutic efficacy *in vitro* and *in vivo* (39, 40).

The initial enthusiasm surrounding TNF-α as a therapeutic was subsequently subdued because of the significant toxicities that occurred after systemic delivery in human subjects. Not only did systemic TNF-α administration cause significant adverse effects, but also it was shown to be largely ineffective at reducing tumor progression. Clinical trials performed in the 1980s and 1990s used TNF-α as both monotherapy and in combination with conventional chemotherapy such as alkylating agents (BCNU, carboplatin) (41) and antineoplastic or antitumor antibiotics (actinomycin D, doxorubicin) (42, 43, 44, 45); however, neither of these trials was able to reach their clinical endpoints since considerable toxicities were observed immediately after delivery, necessitating cessation of its administration (46, 47, 48). Even in trials where administered quantities of TNF-α in the periphery were low and approximated endogenous levels, significant adverse effects were observed, including hypotension, metabolic acidosis, thrombocytopenia, leukopenia, neurotoxicity, respiratory arrest, hyperglycemia, and hyperkalemia (46). TNF-α was also shown to cause cachexia, a metabolic condition characterized by loss and wasting of skeletal muscle, anemia, reduced caloric intake, and altered/decreased immune function (49), leading to TNF-α acquiring the nickname of “cachexin.” At this time, there are no drugs approved for the prevention or treatment of cachexia, only supportive care in terms of dietary modifications, physical therapy, and, in extreme cases, the use of systemic corticosteroids (50). Phase II clinical studies of TNF-α as monotherapy for the treatment of cancer were similarly disappointing. While these focused on advanced and metastatic cases of disease, no complete responses were observed, partial responses were rare, and significant adverse events were also common. Given the lack of efficacy and the substantial toxicities that subjects experienced, these studies significantly dampened previous enthusiasm for TNF-α as a means of tumor control (38, 51, 52, 53, 54, 55, 56, 57).

To circumvent the immune-related adverse events observed following TNF-α therapy, different attempts were made to decrease its toxicity by genetically modifying the cytokine or by localizing the delivery in order to prevent systemic effects. Some groups turned to isolated limb perfusion to localize and concentrate the effects of TNF-α in patients with melanoma or sarcoma (58, 59). In addition, other groups turned to oncolytic viruses encoding transgenic TNF-α to localize its delivery (35, 60). Despite efforts to localize the cytokine to achieve maximum efficacy with minimal toxicity, studies still showed no significant impact on tumor growth, along with significant adverse events, raising the question of whether TNF-α is a viable anticancer agent. Much research has been done in this regard, which demonstrated certain scenarios where TNF-α can act as both a pro- or antitumor agent, depending not only on the timing of administration but also on the context of the malignancy.

## TNF-α as a pro- and antitumor agent

**Figure 1:**
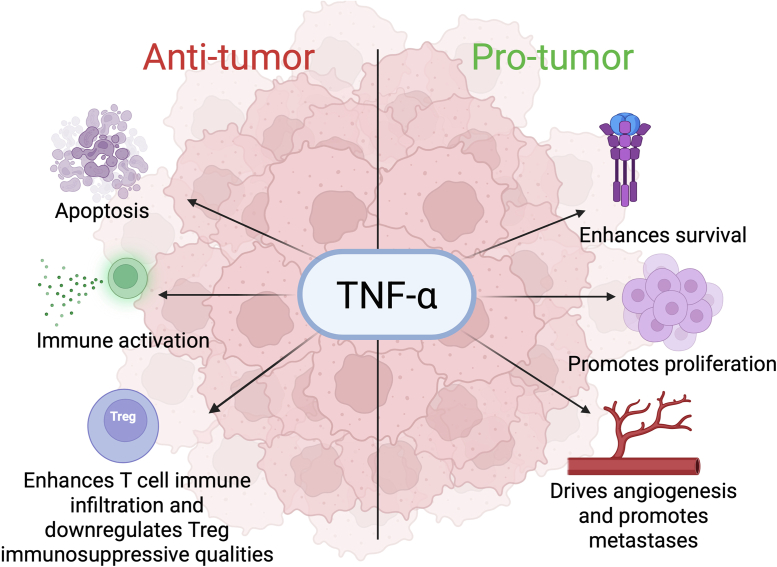
**TNF-α can act in both antitumor and protumor pathways.** While TNF-α has mostly been associated with being antitumor because of its ability to be directly cytolytic, enhance immune infiltration into the tumor, and downregulate immunosuppressive cell activity, it has also been discovered to be implicated in enhancing protumor activity. TNF-α can enhance survival of tumor cells directly by triggering canonical NF-κB signaling and promoting prosurvival gene expression. It can also enhance proliferation of cancer cells directly and drive angiogenesis, which can promote metastasis by facilitating travel of tumor cells to peripheral organs. In this context, TNF-α plays a double-edged sword for cancer. TNF-α, tumor necrosis factor alpha.

Despite the initial evidence that TNF-α, as it was aptly named, would primarily cause necrosis of tumors, preclinical and clinical studies observed more of a double-edged sword in which TNF-α could both promote tumor clearance and advance tumor progression (Fig. 1). Primarily, by virtue of TNF-α being a potent proinflammatory cytokine, chronic release of TNF-α can contribute to tumorigenesis. In addition, more aggressive forms of malignancy, particularly breast cancer, have been characterized by having higher levels of TNF-α, and individuals with lower levels of systemic TNF-α responded better to therapy (61, 62). These data are supported by the preclinical studies that have confirmed the ways in which TNF-α can significantly change the tumor microenvironment (TME) to drive tumor progression. From promoting proliferation directly, enhancing angiogenesis, recruiting immunosuppressive cell types to the TME, and driving local invasion and distant metastasis, TNF-α can potently increase the aggressiveness of disease (63, 64).

As previously discussed, the anticancer qualities of TNF-α are primarily its direct cytotoxicity. TNF-α can trigger cell death by triggering signaling cascades after engaging with TNFR1. However, TNF-α can also induce necrotic cell death *via* activation of NADPH oxidase, leading to the generation of reactive oxygen species. Despite this cytokine having the ability to trigger cell death directly, subsequent research has shown that the main mechanism by which TNF-α achieves any antitumor efficacy is by recruiting and activating other cell types.

Differential expression of the two TNF-α receptors present within the tumor can affect what role TNF-α plays in terms of being protumor or antitumor. High TNFR1 expression on tumor cells is most commonly associated with a “tumor suppressor” environment where TNF-α binding to TNFR1 can lead to cell death directly. However, when there is high expression of TNFR2 on tumor cells, as is the case for certain types of malignancy, including glioblastoma, kidney renal clear cell carcinoma, lower grade brain glioma, pancreatic adenocarcinoma, stomach adenocarcinoma, and testicular germ cell tumors, this environment is more suggestive of being “tumor promoter” since abundant TNFR2 signaling can enhance survival and proliferative pathways (65).

## TNF-α and its modulation of T cells

TNF-α plays a vital role in enhancing T-cell responses, particularly within the TME. TNF-α has been shown to promote CD8+ T-cell infiltration of tumors, commonly correlated with improved outcomes and responses to therapy since these cells are directly cytotoxic (66). However, TNF-α has also been shown to promote the clonal expansion of activated effector T cells and enhance activation of naive T cells, potentially by virtue of both canonical (survival and function) and noncanonical NF-κB activation (enhances T-cell activation and expansion) (67, 68). Since antigen-mediated TCR activation only weakly activates NF-κB activation, TNF-α has been suggested to also act as a costimulatory signal to not only enhance TCR activation directly but also to lower the threshold necessary to initiate TCR signaling (69, 70). Altogether, evidence has shown that this potent proinflammatory signal not only improves T-cell infiltration into the tumor but also significantly enhances T-cell function. This is in comparison to the dual role it plays in Tregs, as it can both downregulate their immunosuppressive qualities, while also facilitating Treg accumulation within the tumor. While TNF-α plays this role on Tregs directly, it also has been reported to have the ability to increase resistance to Treg-mediated suppression (71).

**Figure 2:**
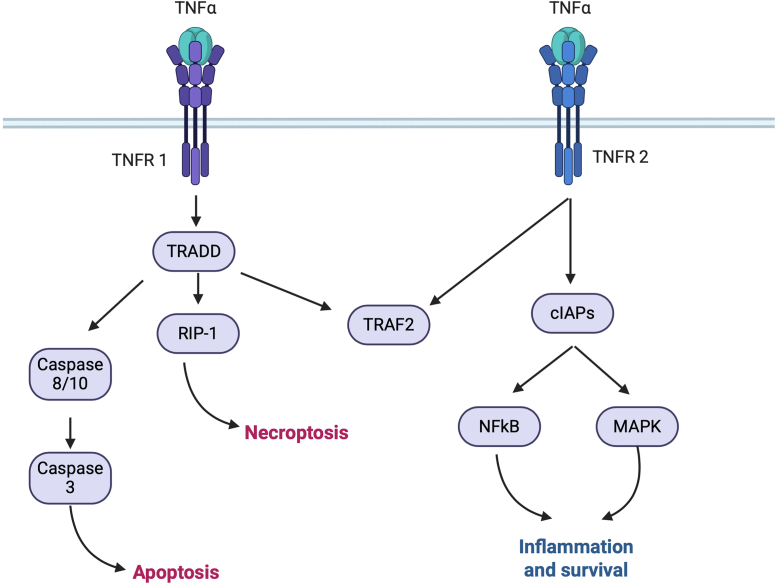
**TNF-α signaling through TNFR1 and TNFR2 in T cells.** TNF-α binding to TNFR1, mostly triggered by soluble TNF, can trigger the assembly of the apoptotic complex composed of TNFR-associated death domain (TRADD) and TNFR-associated factor 2 (TRAF2), which can trigger the RIP kinase pathway leading to necroptosis or lead to caspase activation leading to apoptotic cell death. Comparatively, engagement of TNF-α to TNFR2, which is mostly triggered by membrane-bound TNF-α, leads to the activation of cellular inhibitors of apoptosis proteins (cIAPs), which will lead to the activation of NF-κB and MAPK gene expression pathways leading to inflammation and survival genes. In the context of T cells, these pathways illustrate the ways in which TNF-α in a tumor can enhance or dampen T-cell responses to either enhance antitumor activity or suppress it. MAPK, mitogen-activated protein kinase; RIP, receptor-interacting protein; TNF-α, tumor necrosis factor alpha; TNFR, tumor necrosis factor receptor.

In terms of survival, TNF-α also plays a dual role when it comes to T-cell activity. While TNFR1 signaling can trigger cell death directly, and T cells containing TNFR1 can die from its engagement of TNF-α, they also contain TNFR2, which is a prosurvival receptor capable of mediating not only survival but also enhancing proliferation (Fig. 2). However, the interplay between both receptors in T cells specifically is highly complex. For example, TNFR1-mediated cell death has been well defined, but recent results have suggested that TNFR2 also has the ability to trigger apoptotic cell death by virtue of TNFR2 signaling recruiting TRAF2–cIAP1/2 complexes, which in turn deplete their presence in the cytoplasm and lower the threshold for noncanonical NF-κB signaling, leading to enhanced TNFR1-induced cell death (32). This mechanism has been studied in macrophages and has not been confirmed to occur in T cells. However, TNF-α has been shown to drive activation-induced cell death (AICD) in activated CD8+ T cells within the TME (72, 73). This phenomenon stems from activated CD8+ T cells dying upon activation because of TNFR2 engagement. While this mechanism was hypothesized to prevent excessive clonal expansion upon exposure to chronic antigen, it has also manifested because of immunotherapies due to the large amount of immune activation taking place. In conjunction with the routine use of checkpoint blockade to prevent T-cell exhaustion, AICD has become more prevalent as a response to this T-cell hyperactivation. While the mechanism itself remains elusive, recent reports confirm the ability of reactivated CD8+ T cells undergoing clonal expansion to reach a threshold of activation and yield to TNFR2-mediated cell killing termed AICD, which further establishes the role of TNF as both an enhancer and attenuator of T-cell–based immune responses (74).

## Transcriptional and translational regulation of TNF-α in the context of malignancy

While a main investigational focus is TNF-α production and secretion from immune cells as a response to malignancy, TNF-α production and release is also severely impacted in a variety of malignant cell types. For example, T-cell–based malignancies often have overexpression or mutations in signaling molecules affecting the NF-κB pathway, which inevitably affect TNF-α production and secretion. Similarly, TNF-α promoter regions can be modified or hypomethylated in some cancers and hematologic malignancies, as well as superenhanced in some T-cell lymphomas, driving high expression of TNF-α and other cytokines (75, 76).

**Figure 3:**
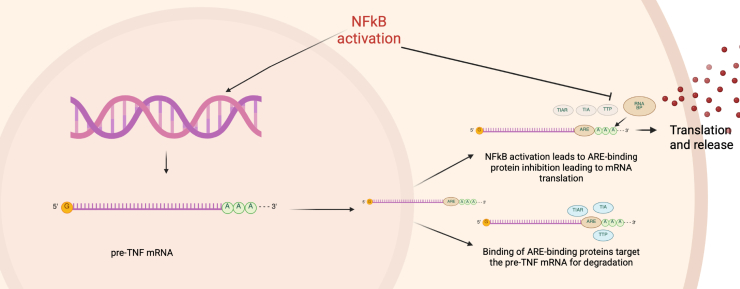
**Regulation of TNF-α.** TNF-α is controlled at both the transcriptional and translational levels. Preformed TNF-α mRNA is released from the nucleus into the cytoplasm, where the availability of ARE-binding proteins determines whether the mRNA is translated to a protein, or if it gets targeted for degradation, which occurs when ARE-binding proteins bind to the AREs. This ensures that there is substantial preformed TNF-α mRNA ready to be translated and released quickly. Post-translational regulation of TNF-α occurs in an activated state, where precursor TNF-α cytokine accumulates within the cell but is not released. Extracellular stimulus, activation status, and signaling cascades regulate the intensity and timing of TNF-α production and release in a well-controlled process. ARE, AU-rich element; TNF-α, tumor necrosis factor alpha.

TNF-α mRNA contains AU-rich elements (AREs) in the 3′ untranslated region that target it for rapid degradation; this is what prevents it from accumulating inside of the cell and what allows for TNF-α to lack mRNA stability. Normally, transacting proteins that bind AREs stabilize or destabilize the transcript depending on the context. Three different ARE-binding proteins have been identified specifically for TNF-α ARE: tristetraprolin, a lipopolysaccharide-inducible protein that destabilizes the TNF-α mRNA, TIAR, and related protein TIA-1, which also bind the ARE, and are related with acting as a translational silencer (77, 78). These ARE-binding proteins are always active, except in the context of activation, which leads to ARE repression and allows the TNF-α mRNA to stabilize in order to be translated and subsequently secreted from the cell (Fig. 3) (79). This pathway, however, has been observed to be dysregulated by affecting specific ARE-binding proteins, which stabilizes TNF-α mRNA and increases protein expression. While this would seem to be detrimental in the context of this malignancy because of the antitumor effects, the hypothesis is that increased TNF-α production leads to NF-κB activation, which facilitates growth and survival. In addition, increased protein expression could be sensitizing these cells to TNF-α expression and release, therefore making them more resistant to immunotherapies or immune-secreted TNF-α in the future (80).

In the context of T cells, transcriptional and translational control of TNF-α production is also tightly regulated and significantly affects the cellular responses that are triggered. For example, CD8+ T cells, in particular, employ several mechanisms by which they drive cytokine production upon T-cell activation. Studies have found large amounts of preformed TNF-α mRNA in resting CD8+ T cells, which can drive rapid translation upon activation by phosphorylation of eukaryotic initiation factor 4E (eIF4E) by mechanistic target of rapamycin, leading to its binding to TNF-α mRNA 5′ structures, which facilitates formation of the preinitiation complex, allowing it to be translated by ribosomes (81, 82). This effect was observed exclusively in activated T cells, whereas in resting T cells without antigenic challenge, eIF4E was primarily expressed in its unphosphorylated isoform, repressing translation of the TNF-α mRNA (83). This means that to mount a potent TNF-α response, activated T cells need incredible post-translational aid in order to translate the preformed TNF-α mRNA both by destabilizing the formed mRNA through its ARE region, and by using PKC-mediated mechanistic target of rapamycin phosphorylation of eIF4E to drive the formation of the ribosome complex (82). This is particularly impactful when we consider the timing of these events and the transient presence of the RNA-binding proteins (RBPs) in mediating cytokine release. For example, studies have shown that in CD4+ T cells, two RBPs prevent them from exiting quiescence by destabilizing mRNAs that encode for differentiation and function (84). Similarly, ZFP36L2 RBP prevents aberrant protein production in resting memory T cells to keep them in a resting state, even though the mRNAs are already formed (85). Reactivated memory T cells rely on ZFP36L2 as well to release them from their resting state and reacquire their ability to produce proinflammatory cytokines. This means that control of RBPs inevitably leads to control over cytokine production as well. More recent studies have shown an interaction between T-cell activation and the competition between RBPs and microRNA binding. In addition, Popovic *et al*. (86) have shown that combined deletion of ZFP36 family proteins can delay the shutdown of cytokine production in T cells in the presence of tumor cells, allowing for enhanced proinflammatory cytokine release, or can boost effector function, which leads into an interesting line of study on how these RBPs can be used therapeutically to regulate these T-cell–based responses.

Altogether, this suggests that post-transcriptional regulation and control of TNF-α mRNA regulates the potency of T-cell responses elicited, and almost as importantly, within the tumor, seem to sensitize tumor cells against TNF-α-mediated killing.

## TNF-α sensitization in the context of immunotherapies

The capacity of TNF as a protumor and antitumor agent could rely on the timing of its activity. Tumor cells can become resistant to TNF-α cytotoxicity through NF-κB transcription leading to increased cell survival or NF-κB–driven resistance to apoptosis. This could also be the reason why anti-TNF-α agents have gained popularity at not only decreasing TNF-α-mediated toxicities but also eliciting prolonged disease stabilization since they bypass this triggering of prosurvival NF-κB signaling (64, 87, 88, 89). Anti-TNF-α therapies (*e.g*., infliximab, etanercept) sequester soluble TNF-α, thus preventing signaling cascades that promote survival. Alternatively, cancer cells with prolonged exposure to TNF-α are sensitized and require another mechanism to trigger apoptosis independent of TNF-α signaling. Another hallmark of chronic TNF-α signaling within the tumor that sensitizes tumor cells is increased recruitment of macrophages, which can increase PD-L1 expression on tumor cells, leading to increased T-cell exhaustion and a decrease in their efficacy (90, 91).

In addition, chronic TNF-α secretion within the tumor leads to the induction of inhibitory cell types, such as MDSCs and Tregs, both of which contribute to dampening antitumor immune responses (92, 93). This could imply that the tumor cells themselves are not being sensitized by the presence of the cytokine, but rather that chronic TNF-α signaling leads to an immunosuppressive TME that inhibits the natural mechanisms of TNF-α-mediated killing. Last, a characteristic of TNF-α that influences the sensitivity of cancer cells to immunotherapy is that prolonged exposure to the cytokine could make cancer cells dependent on TNF signaling for survival, therefore negating benefit from TNF-α secretion by immune cells into the tumor and bypassing TNF-α-mediated cell killing (94). At this point, sensitization would have progressed into being dependent by the cancer cells on the cytokine to grow and survive.

What becomes more evident is the pattern of balance when it comes to TNF-α. Early secretion of TNF-α sensitizes tumor cells to TNF-α-mediated killing by binding to TNFR1, killing them directly. TNF-α can also cause necroptosis through the RIP kinase pathway (95). However, too much TNF-α within the tumor leads to NF-κB resistance to apoptosis (96). Similarly, localized TNF-α within the tumor sensitizes tumor cells to immune attacks and chemotherapy. However, too much TNF-α (as occurs in chronic inflammation) can lead to tumorigenesis and promote restructuring of the TME to promote metastasis (64). This suggests that acute exposure to TNF-α, in early phase immune response to cancer, generally promotes immune cell activation and tumor cell death. However, chronic exposure to TNF-α, particularly in established tumors, normally leads to exhaustion, immune suppression, and enhances resistance mechanisms (97). This might be difficult to exploit therapeutically, considering that TNF-α′s systemic responses build with time. Therefore, high levels of TNF-α within the TME could create a vicious cycle, whereby tumor cells die, eventually evade immune attack, and lead to eventual dampening of the immune response by prolonged TNF-α exposure. One way to circumvent this is to block the NF-κB pathway to prevent sensitization of cells to the effects of TNF-α prior to immune activation. Actinomycin D and flavopiridol can be used as inhibitors of transcription, and topoisomerase II inhibitors can also be administered to increase sensitivity to TNF-α-mediated killing of resistant tumor cells (38).

In a therapeutic setting, TNF-α blockers are used for treatment of a wide variety of inflammatory disorders, although much more work is necessary to understand the role these inhibitors play in cancer progression. Whether these TNF-α blocking agents can synergize with conventional immunotherapies or be complementary to the standard of care remains an open question. These therapies, however, come with many unexplored phenomena. While they might be helpful in alleviating chronic TNF-α exposure, while retaining the initial benefit of acute TNF-α release within the tumor, little is known about the effects of long-term use of TNF-α inhibitors. There are a variety of Food and Drug Administration–approved TNF-α blockers, ranging from monoclonal antibodies, secreted forms of TNF-α receptors, and even PEGylated anti-TNF-α agents. However, the question remains on whether administering these TNF-α blockers could enhance tumor progression long term, or if they themselves would promote cancer initiation. Recent studies measuring cancer risk in adults with rheumatoid arthritis taking TNF-α inhibitors showed that TNF-α inhibition was significantly associated with increased risk for certain malignancies, such as follicular lymphoma, non-Hodgkin’s lymphoma, and nonmelanoma skin cancer (98, 99). In contrast, many other studies have not shown an increased risk for cancer development after TNF-α inhibitor use, even in patient populations with different inflammatory disorders (100, 101, 102, 103, 104). Further research is necessary to understand the causative relationship between these therapies, or if the increased risk is simply because of the pre-existing inflammatory disease, creating a chronic inflammatory state, which can facilitate transition to a malignant state (104, 105, 106).

## Molecular switches and cellular context that influence TNF as a tumor suppressor/promoter

Understanding when TNF-α can act as a tumor suppressor *versus* a tumor promoter is important, but of equal importance is to assess what types of therapies can synergize with TNF-α activity or instances in which we can differentiate TNF-α accumulation as being beneficial from detrimental. Early on, when TNF-α is acting as a tumor suppressor and initiating cancer cell killing, it would be appropriate to combine it with sensitizers, such as SMAC (second mitochondrial-derived activator of caspases) mimetics inhibitors and inhibitors of apoptosis protein (IAPs) to maximize cell killing (107). In addition, permissive enhancement of TNF-α signaling during initial T-cell immunotherapy, such as checkpoint blockade, could boost immune killing (108). Comparatively, in the context of large amounts of TNF-α already present in the tumor and in a chronic exposure setting, inhibitors of NF-κB signaling would ensure cells stay vulnerable to TNF-α-mediated killing, and in other cases, stopping TNF-α altogether would be more appropriate, in which case, anti–TNF-α therapies can be used (109). In this context, lowering TNF-α concentrations can also be impactful if patients experience immune-related adverse events during checkpoint therapy or other forms of immunotherapy suggestive of damaging amounts of TNF-α within the tumor leading to systemic toxicities.

**Figure 4:**
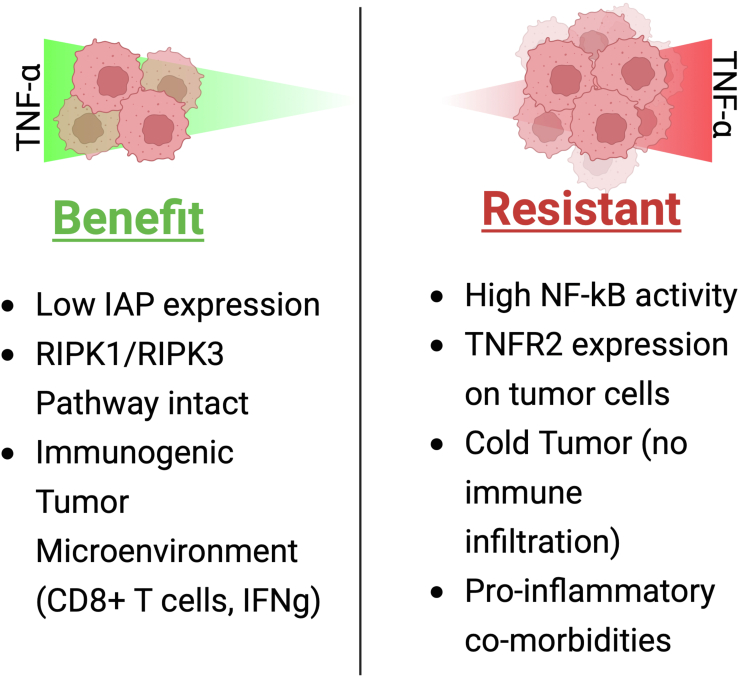
**Context in which TNF-α therapy could be beneficial.** Tumor components influence whether TNF-α can act in a protumor or antitumor manner. As such, tumors that have high immune infiltration and contain qualities that enhance the assembly of the proapoptotic pathway would benefit from TNF-α therapy since these pathways will enhance tumor cell death directly. However, if the tumor has already been sensitized to TNF-α-mediated killing leading to canonical NF-κB activation, expresses surface receptors that enhance prosurvival pathways, such as TNFR2, and overall lacks immune infiltration by which TNF-α-mediated therapies enhance tumor cell killing, these factors can make the tumor microenvironment resistant to TNF-α-based therapies. It is important to also note that proinflammatory comorbidities might not make tumors resistant to TNF-α-based therapies, but they would augment the risk for immune-related adverse events, which are commonly a limiting factor for these therapeutics. TNF-α, tumor necrosis factor alpha; TNFR, tumor necrosis factor receptor.

Predictive factors for TNF-α activity as a tumor promoter or a suppressor include post-translational modification of key factors that affect the TNF-α signaling pathways, such as ubiquitination of RIPK1, which inherently activates the NF-κB prosurvival pathway (Fig. 4) (110). The presence of IAPs can also be a good negative predictor for efficacy, since they will innately promote cell survival by preventing apoptosis (111). Predictive factors for TNF-α being a tumor suppressor include having higher expression of TNFR1 present on tumor cells, which can lead to higher levels of TNF-mediated cytotoxicity, although some studies have shown that increased TNFR1 presence has been correlated to negative prognosis for some cancer models (64). In addition, deubiquitination of RIPK1 can be predictive of higher TNF-α killing because of the RIP kinase pathway promoting death signaling. These might be good indicators by which one can categorize which patients would benefit from certain immunotherapies that inherently produce large amounts of TNF-α, and in which cases these therapies might cause toxicities or would be detrimental. In addition, these predictive factors better inform the ways in which TNF-α can be fully harnessed to elicit higher amounts of killing without the side effects. For example, patients who have tumors with low IAP expression, or show responsiveness to SMAC-mimetics, would suggest that they would benefit from therapies that elicit high levels of TNF-α or from TNF-α–based therapies. Considering that SMAC mimetics trigger caspase activation directly, this environment would synergize with TNF-α-based therapies, considering that the TNF-α produced will only enhance the already active proapoptotic pathways that are triggered (112). In addition, ensuring that the RIPK1 and RIPK3 pathways are intact would be important for TNF-α to trigger necroptosis, especially since it is a pathway commonly altered during malignancy (113). A third compartment would be characterizing the TME to ensure that TNF-α can synergize with the immune responses taking place, in which case a TME with high immune infiltration of CD8+ T cells, low Tregs, and high levels of other proinflammatory cytokines such as interferon-gamma would benefit this treatment regimen (114). Ensuring that the tumor cells express low activity of p65, ReIA, and NF-κB would also help, given that they would be less susceptible to TNF-α-mediated survival pathways (115). While some tumor types are inherently more responsive to localized TNF-α–based therapies, such as certain sarcomas, melanomas, and highly vascularized tumors, it is important to derive a holistic view of characteristics that might make some models or some individuals more sensitive to TNF-α–based therapies (116).

Conversely, there are certain demographics of patients that would not benefit from TNF-α–based therapies. If the tumor expresses high levels of ReIA, p65, has high infiltration of immunosuppressive cell types, such as Tregs and MDSCs, overall lacks immune infiltration or has very low antigenic burden, and exhibits dysregulation of RIPK pathways, these scenarios compromise the integrity of the pathways TNF-α uses to cause cytotoxicity or apoptosis. Such cases would therefore not benefit from TNF-α–based therapies (13, 117, 118). On the contrary, introducing high levels of TNF-α might drive such tumors to increased survival, invasion, and metastatic phenotypes. In addition, patients who experience previous comorbidities such as autoimmune disease might not benefit from TNF-α–based therapies considering the high threshold of inflammation that might have sensitized their cancer cells already, making them less susceptible to killing.

Some tools we can use to confirm these results and stratify patients include RNA-Seq and gene expression profiling for the presence or the absence of certain genes and TNF-α targets. In addition, one can use flow cytometry or immunohistochemistry to quantify the abundance of certain cell surface receptors such as TNFR1 and TNFR2. In addition, we can use clinical biomarkers such as C-reactive protein and interleukin-6 to understand the inflammatory state of patients prior to starting TNF-α–based therapies for their tumors (119).

## Conclusion

TNF-α is a well-studied pleiotropic cytokine with influence in cellular processes that extend to everything from growth and survival to cell death. This review has summarized the cellular processes and molecular switches that influence whether tumors are more or less susceptible to TNF-α–mediated killing. There are several biomarkers that one can consider whenever determining if a patient would respond to TNF-α–based therapies, mainly surrounding controlling the activation of NF-κB, cIAPs, and the endogenous immune infiltration present in the tumor. Comparatively, there are ways to infer whether a patient with cancer would not benefit from TNF-α therapy and would be at higher risk for immune-related adverse events. These include previous autoimmune conditions, the abundance of TNFR2 on the surface of cells, and the presence of immunosuppressive cells within the tumor.

We also explored topics centralized on whether the timing of TNF-α delivery would enhance or diminish immune activity. Previous studies show that acute TNF-α activation is significant in enhancing cell-based immunotherapy activity and synergizes with immune checkpoint blockade, two therapies routinely used in patients today. However, chronic exposure to TNF-α, which would happen after prolonged immune activation within the tumor or because of an endogenous amount of TNF-α within the tumor already, would prove to be detrimental to these therapies. Moreover, TNF-α in this context would be harmful and would potentially act as a tumor promoter, driving survival, angiogenesis, and metastatic phenotypes.

Overall, while TNF-α remains a double-edged sword in its role in cancer progression and regression, previous and ongoing research has helped refine the instances in which one can understand the nuances of TNF-α–based cancer therapies, particularly in the context of localized delivery with new and emerging technology.

## Conflict of interest

T. S. N. reports consulting honoraria from Allogene Therapeutics, PACT Pharma, Adaptive Biotechnologies, and Medidata Solutions.
